# Oxygenated deep bottoms beneath a thick hypoxic layer lack potential of benthic colonization

**DOI:** 10.1007/s13280-017-0938-2

**Published:** 2017-08-19

**Authors:** Anders Stigebrandt, Rutger Rosenberg, Marina Magnusson, Torsten Linders

**Affiliations:** 1 Department of Marine Sciences, University of Gothenburg, Box 461, 40530 Göteborg, Sweden; 2 Department of Biology and Environmental Sciences, University of Gothenburg, Kristineberg 566, 45178 Fiskebäckskil, Sweden; 3 Marine Monitoring AB, Strandvägen 9, 45330 Lysekil, Sweden

Comment to: Stigebrandt, A., B. Liljebladh, L. De Brabandere, M. Forth, Å. Granmo, P.O.J. Hall, J. Hammar, D. Hansson, et al. 2015: An experiment with forced oxygenation of the deepwater of the anoxic By Fjord, western Sweden. *Ambio* 44: 42–54. doi:10.1007/s13280-014-0524-9.

## Background

**Fig. 1:**
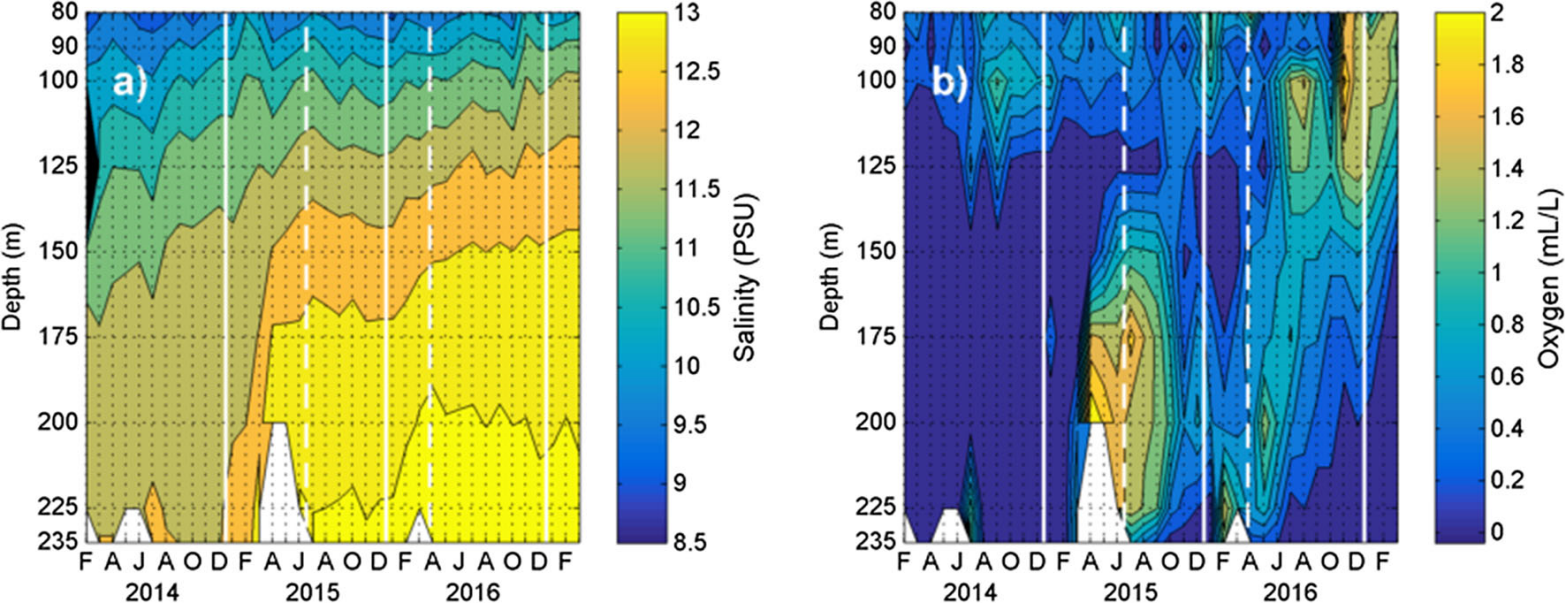
Monthly observations by SMHI (the Swedish Meteorological and Hydrological Institute, https://sharkweb.smhi.se/) at BY15 in the East Gotland basin: **a** salinity and **b** oxygen. *Letters* below panels indicate the middle of every second month. Observations from May 2014, May and August 2015, and June 2016 were unavailable and interpolated values are used. *Solid white lines* mark year endings; *dashed white lines* mark timing of SPI expeditions

**Fig. 2:**
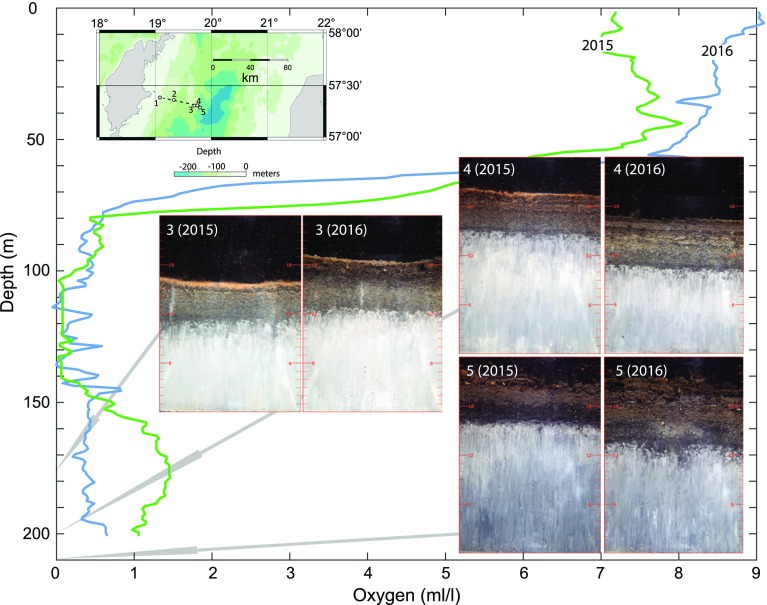
Sediment profile images (size 17 × 24 cm) obtained at Stations 3, 4 and 5 in April 2016. Also shown are images from July 2015 (from Rosenberg et al. 2016). Oxygen depth profiles from these dates are also shown

After many years with mainly anoxic conditions, the deepwater in the East Gotland Basin (EGB) in the Baltic became oxygenated when a huge inflow of new deepwater from the Kattegat in December 2014 reached the EGB (e.g. Mohrholz et al. 2015). During such inflows, the rather saline and dense new deepwater intrudes along the seabed. Some of the residing old deepwater was mixed into the new deepwater, which successively reduced both the salinity and the oxygen concentration of the intruding new deepwater. The unmixed portion of the old deepwater was lifted over the new deepwater. In March 2015, about 3 months after the inflow from the Kattegat, the new deepwater reached station BY15 in the EGB as can be seen from the strong increase of salinity (Fig. 1a) and the appearance of oxygen (Fig. 1b). BY15 is located ca 10 km east of Station 5 (Fig. 2).

On 2 July 2015, about 4 months after the oxygenation of the deep bottoms in the EGB, Rosenberg et al. (2016) took sediment profile images (SPI) of the upper part of the sediment at Stations 1 to 5, located at 60, 140, 175, 200 and 210 m depth, respectively. Station 1 is a reference station located at the top of the halocline above the deepwater. The images showed thin oxidized layers at the sediment surface at all stations except Station 2 (Rosenberg et al. 2016). The chemical conditions in the top sediments in 2015 are described in that paper in relation to the redox conditions. The sediment images showed signs of macroscopic life only at shallow Station 1 where signs of bioturbation by sediment-dwelling animals (infauna) were observed. The lack of signs of animals on the deepest stations, 3 to 5, was believed to be due to a too brief time of exposure to oxygenated water above the sediment. In an earlier engineering experiment, with forced oxygenation of the usually anoxic deepwater in the By Fjord on the Swedish west coast, fauna was established about one year after oxygenation (Stigebrandt et al. 2015).

To follow the progress of possible colonization and the progress of chemical oxygenation and reduction processes of the bottoms overlain by hypoxic water in the deeper EGB, a SPI survey similar to the July 2015 survey of Rosenberg et al. (2016) was undertaken on 10 April 2016. The results of these observations are shown and discussed below. A long-term historic development of oxygen records of the Baltic and the Gotland Basin is given by Conley et al. (2009).

## Results and discussion

In April 2016, about one year after the oxygenation of the deeper bottoms in the EGB, much of the oxygen supplied by the inflow that began December 2014 had been consumed. At Station 2, 140 m depth, the oxygen concentration had been quite low for a few months before the SPI survey. The oxygen reduction was similar at Station 3 at 175 m depth (Fig. 1b). Below 200 m depth, the oxygen content increased in February 2016 after having been quite low before this. The simultaneous increase in salinity suggests that this event was due to a new inflow from the Kattegat a couple of months earlier.

For comparison, images from 2015 and 2016 are shown together (Fig. 2). Bioturbating animals were not observed in the 2016 images. Oxygen concentrations in relation to depth from July 2015 and April 2016 are shown in Fig. 2. A dramatic decline in oxygen was recorded both years in the halocline from about 50 to 80 m water depth reaching less than 0.7 mL L^−1^. In 2016, this low concentration prevailed down to 200 m, whereas in 2015 it increased to slightly above 1 mL L^−1^ from 160 to 200 m.

At station 3 (175 m deep), the yellow surface layer indicative of manganese and iron oxides had become thinner from 2015 to 2016 (Fig. 2). This was likely related to recent declines in oxygen concentration (Fig. 1b). Station 4 (200 m deep) had a thin oxidized layer at the sediment surface in 2015, but this had been reduced and almost disappeared in 2016. Station 5 (210 m deep) showed only minor and scattered signs of oxidation for both years. Rosenberg et al. (2016) documented low oxygen concentrations close to zero between 100 and 140 m depth in 2015, which were insufficient to support oxidation of sediment surface layers. Oxygen concentrations around or above 1 mL L^−1^ seemed needed to initiate the oxygenating process and probably also for maintaining such conditions. Hypoxic conditions that significantly impact benthos begin at 2.0 mL L^−1^ of oxygen and extend down to 0.0 mL L^−1^, the point of anoxia (Diaz and Rosenberg 1995). These authors concluded that hypoxia leads to mortality and delayed recolonization of benthos but some tolerant species will survive at concentrations around 1 mL L^−1^ for some time. Pelagic larvae of most species appear to be more sensitive to low oxygen concentration compared to adult species. Succession of benthic communities in the Baltic in relation to hypoxic conditions has been analysed by Rumohr et al. (1996).

**Fig. 3:**
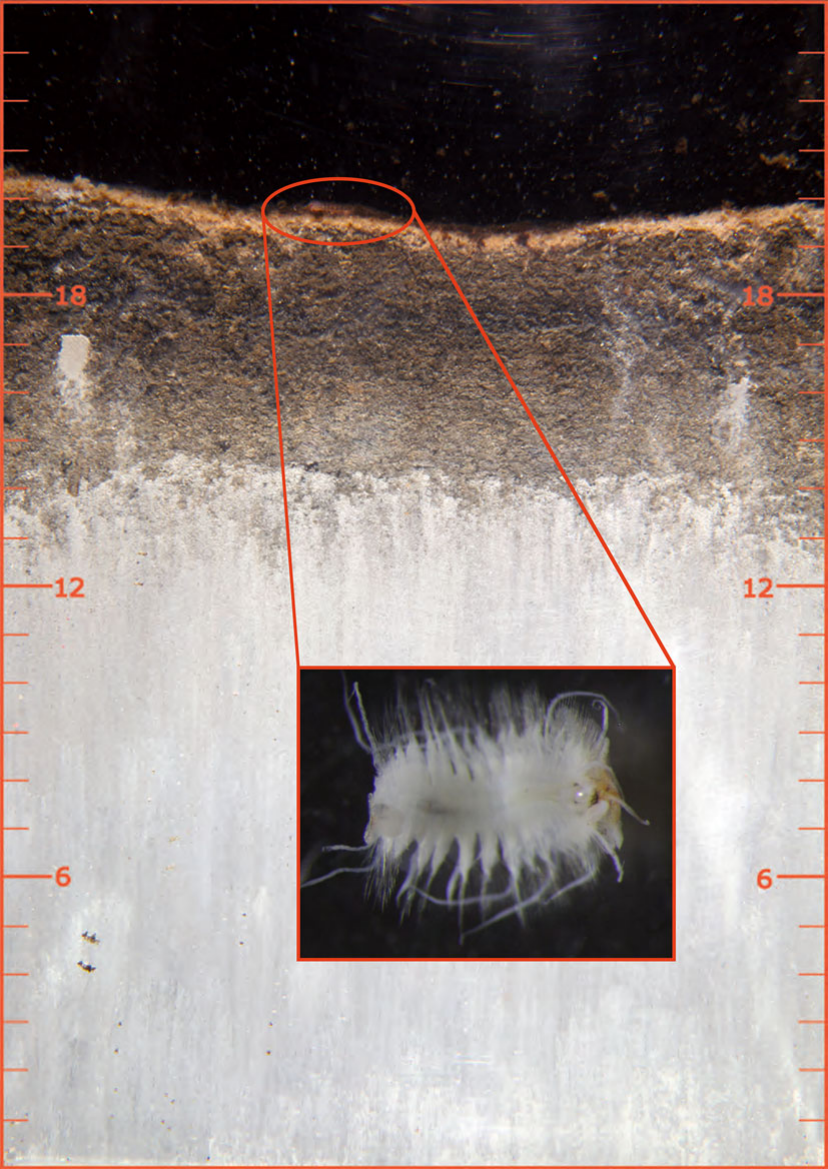
SPI from Station 3 at 175 m depth, 10 April 2016, showing a surface-dwelling polychaete *Bylgides (Harmothoe) sarsi*. *Inset* shows an example of this polychaete

One record of a benthic individual occurred at Station 3 at 175 m depth. This was a sediment surface-dwelling polychaete *Bylgides (Harmothoe) sarsi* (Fig. 3), one of the most common benthic animals around Gotland in more shallow waters (Karlson et al. 2002). No indication of burrowing or recruitment in the sediment was observed in the present study. This suggests that the colonizing possibilities were limited because of hypoxic conditions between 100 and 140 m water depths. Colonization through transport in the incoming water near the bottom was another recruitment possibility.

Unlike the situation in the By Fjord (Stigebrandt et al. 2015), there were no signs of bioturbating animals in the deepwater. The reason for this is believed to be the fact that the layer that became oxygenated in March 2015 was overlain by an about 20-m-thick layer, centred at about 125 m depth, of old deepwater with no or only hypoxic concentrations of oxygen (Fig. 1b). This layer could act as a barrier preventing larvae or adult animals from reaching the bottom vertically. Colonization of the deep bottoms of oxygenated layers that are covered by a thick layer of anoxia or hypoxia can probably best be performed by horizontal movement of adults or larvae carried by the inflowing new deepwater.
